# Role of a lower cutoff of high sensitivity troponin I in identification of early cardiac damage in non-severe patients with COVID-19

**DOI:** 10.1038/s41598-022-06378-2

**Published:** 2022-02-11

**Authors:** Yiting Lin, Kun Yan, Lingling Chen, Yiqun Wu, Jielan Liu, Yingying Chen, Bingbo Hou, Ping Zhong

**Affiliations:** 1 Department of Respiratory and Critical Care Medicine, Xiamen Haicang Hospital, Xiamen, China; 2 Department of Respiratory Section II, The Third Hospital of Xiamen Affiliated to Fujian University of Traditional Chinese Medicine, Xiamen, China; 3 Department of Internal Medicine, Xiamen Lotus Hospital, Xiamen, China; 4 Department of Cardiac and Cerebral Function, Xiamen Xian Yue Hospital, Xiamen, China; 5 Department of Cardiology, Xiamen University Zhongshan Hospital, No.201-209 Hubinnan Road, Xiamen, 361003 Fujian People’s Republic of China; 6 BE and Phase I Clinical Trial Center, The First Affiliated Hospital of Xiamen University, School of Medicine, Xiamen University, NO.55 Zhenhai Road, Xiamen, 361002 Fujian People’s Republic of China

**Keywords:** Biomarkers, Cardiology, Diseases, Pathogenesis

## Abstract

Cardiac damage in non-severe patients with coronavirus disease 2019 (COVID-19) is poorly explored. This study aimed to explore the manifestations of cardiac damage at presentation in non-severe patients with COVID-19. In this study, 113 non-severe patients with COVID-19 were grouped according to the duration from symptoms onset to hospital admission: group 1 (≤ 1 week, *n* = 27), group 2 (> 1 to 2 weeks, *n* = 28), group 3 (> 2 to 3 weeks, *n* = 27), group 4 (> 3 weeks, *n* = 31). Clinical, cardiovascular, and radiological data on hospital admission were compared across the four groups. The level of high sensitivity troponin I (hs-cTnI) in group 2 [10.25 (IQR 6.75–15.63) ng/L] was significantly higher than those in group 1 [1.90 (IQR 1.90–8.80) ng/L] and group 4 [1.90 (IQR 1.90–5.80) ng/L] (all *P*_bonferroni_ < 0.05). The proportion of patients who had a level of hs-cTnI ≥ 5 ng/L in group 2 (85.71%) was significantly higher than those in the other three groups (37.04%, 51.85%, and 25.81%, respectively) (all *P*_bonferroni_ < 0.05). Compared with patients with hs-cTnI under 5 ng/L, those with hs-cTnI ≥ 5 ng/L had lower lymphocyte count (*P* = 0.000) and SpO_2_ (*P* = 0.002) and higher CRP (*P* = 0.000). Patients with hs-cTnI ≥ 5 ng/L had a higher incidence of bilateral pneumonia (*P* = 0.000) and longer hospital length of stay (*P* = 0.000). In conclusion, non-severe patients with COVID-19 in the second week after symptoms onset were most likely to suffer cardiac damage. A detectable level of hs-cTnI ≥ 5 ng/L might be a manifestation of early cardiac damage in the patients.

## Introduction

Coronavirus disease 2019 (COVID-19), caused by severe acute respiratory syndrome coronavirus-2 (SARS-Cov-2), has impacted health globally on an unprecedented scale, and the number of cases continues to rise worldwide^1^. Whereas COVID-19 is primarily known as a respiratory infection, it has important systemic effects such as cardiac damage, which was shown to be a significant contributor to the fatal outcomes of patients with COVID-19^2,3^. Moreover, COVID-19 patients with premorbid cardiovascular diseases were more likely to develop severe symptoms and represented a large proportion of deaths from COVID-19^4^. Therefore, cardiac damage should be especially noticed for patients with COVID-19, especially for patients with premorbid cardiovascular diseases.

To date, the manifestations of cardiac damage, including acute cardiac injury, myocarditis, arrhythmia, heart failure, and cardiogenic shock, have been noted in several published reports on COVID-19^5–9^. However, the majority of them focused on the manifestations of cardiac damage in severe patients with COVID-19, whereas the manifestations in non-severe patients are poorly explored. Since over 80% of COVID-19 patients are non-severe, there is an urgent need to investigate the manifestations of cardiac damage in non-severe patients with COVID-19.

In general, a higher level of hs-cTnI than the 99th percentile is recommended to identify cardiac injury in clinical practice. However, the cutoff of hs-cTnI for effectively predicting mortality of patients with COVID-19 was found to be much lower than the 99th percentile^10^. Moreover, the cardiac involvement, which was revealed by cardiovascular magnetic resonance, was reported in 78% of COVID-19 patients (78/100), whereas only 5% of these patients presented a higher level of hs-cTnI than the 99th percentile^11^. All these results indicated that cardiac injury might be the tip of the iceberg in the cardiac damage of COVID-19. Furthermore, a much lower hs-cTnI cut-off of 5 ng/L has been shown to successfully identify patients with suspected acute coronary syndrome who were at high risk of cardiac ischemic events^12,13^. Accordingly, we hypothesized that a higher level of hs-cTnI than 5 ng/L is one of the manifestations of cardiac damage in non-severe patients COVID-19. To verify this hypothesis, this study aimed to investigate the manifestations of cardiac damage at presentation in non-severe patients with COVID-19.

## Methods

### Study population

The study population was collected from the Wuhan Tongji Hospital Guanggu Branch, Huazhong University of Science and Technology, which was managed by a multidisciplinary team from Xiamen city. The inclusion criteria of the study population were as follows: (1) Consecutive COVID-19 patients admitted to the E3-9 ward in this hospital between February 10, 2020, and March 25, 2020. (2) Based on the “diagnosis and treatment guideline for COVID-19 of China” (http://www.nhc.gov.cn/), patients included in this study were confirmed by detecting SARS-CoV-2 RNA in pharyngeal swab samples. (3) The severity of patients on hospital admission was categorized as non-severe type. According to the guideline, the severe type was characterized by (a) dyspnea (respiratory frequency ≥ 30 rates per minute); (b) blood oxygen saturation ≤ 93%; (c) PaO2/FiO2 ratio < 300, and/or lung infiltrates > 50% within 24–48 h (satisfying at least one of the above items). Non-severe patients included patients with non-pneumonia and mild to moderate pneumonia and satisfied none of the above items.

The exclusion criteria of the study population were as follows: (1) Patients without detailed medical records on hospital admission within 24 h (e.g., without cardiac biomarkers); (2) Patients who had a history or the presence of myocardial infarction and heart failure; (3) The date of the illness onset had not been recorded accurately. All methods were carried out following the Declaration of Helsinki. The Ethics Committee of Xiamen Haicang Hospital approved this study under an expedited review. Meanwhile, informed consent was waived by the Ethics Committee of Xiamen Haicang Hospital due to this retrospective design.

### Study design

Initially, we intended to explore the period when COVID-19 patients were most likely to suffer cardiac damage. According to the previous theories^14–16^, we designated four groups of patients based on the duration from symptoms onset to hospital admission: group 1 (admitted to the hospital within one week), group 2 (admitted to the hospital > one week to two weeks), group 3 (admitted to the hospital > two weeks to three weeks), group 4 (admitted to the hospital > three weeks).

Demographic information and clinical medical records from COVID-19 patients on hospital admission within 24 h were extracted. Clinical characteristics included symptoms onset, the duration from symptoms onset to hospital admission, vital signs on hospital admission, comorbidities, medications for the treatment of comorbidities, laboratory indices (e.g. lymphocyte, creatinine, urea, C-reactive protein (CRP), cardiac biomarkers, etc.) and radiological findings. In this study, high sensitivity troponin I (hs-cTnI) was measured by using a high sensitivity assay (ARCHITECT STAT, Abbott Laboratories) at the clinical laboratory of Tongji Hospital. According to the manufacturer, the 99th percentile concentrations are 34.2 ng/L for males and 15.6 ng/L for females, with a corresponding coefficient of variation of < 5%. The extracted data were compared across the four groups. In addition, the levels of CRP, lymphocyte count, pulse oximeter O_2_ saturation (SpO_2_), cardiac biomarkers, and lung involvement, and hospital length of stay were compared between patients with hs-cTnI under 5 ng/L and ≥ 5 ng/L.

### Statistical analysis

The data were analyzed by SPSS statistic 22.0 (SPSS Inc., Chicago, USA). Continuous variables were expressed as the median and interquartile range (IQR) when they were highly skewed distribution, and the differences were analyzed using independent samples Kruskal–Wallis test or Mann–Whitney U test, as appropriate. Categorical values were expressed as frequencies, and the differences were analyzed using χ^2^ tests or Fisher’s exact test across the four groups. Correlations between cardiac biomarkers and SpO_2_, lymphocyte count, and CRP were tested by Spearman correlation analyses. Besides, a boxplot (without outliers) was drawn in the case of a significant difference for this indicator across the four groups. The critical *P* value (α) was defined as 0.05, and all statistical significance was defined as *P* < 0.05. However, the *P* value was adjusted using the Bonferroni method when a pairwise comparison in the four groups was conducted.

## Results

### Comparisons of demographic and clinical characteristics across the four groups

In this study, we enrolled 113 non-severe patients with COVID-19 finally. Comparisons of demographic and clinical characteristics across the four groups are shown in Table 1. There were no significant differences in age, sex, comorbidities, medications, symptoms, creatinine, urea, and hospital length of stay across the four groups. Of note, the level of SpO_2_ in group 2 was significantly lower than those in group 1 and group 4 (all *P*_bonferroni_ < 0.05).

**Table 1:** Comparisons of demographic and clinical characteristics across the four groups.

Characteristics	Total (*n* = 113)	Group 1 (*n* = 27)	Group 2 (*n* = 28)	Group 3 (*n* = 27)	Group 4 (*n* = 31)	H/χ^2^	*P*
**Age (years)**	57.0 (43.5–69.0)	54.0 (35.0–69.0)	61.0 (52.5–70.0)	64.0 (41.0–69.0)	54.0 (45.0–67.0)	2.438	0.487
< 60	61 (53.98)	18 (66.67)	12 (42.86)	12 (44.44)	19 (61.29)	4.799	0.187
≥ 60	52 (46.02)	9 (33.33)	16 (57.14)	15 (55.56)	12 (38.74)		
**Sex**							
Male	59 (52.21)	13 (48.15)	16 (57.14)	15 (55.56)	15 (48.39)	0.754	0.860
Female	54 (47.79)	14 (51.85)	12 (42.86)	12 (44.44)	16 (51.61)		
**Comorbidity**							
Hypertension	34 (26.55)	5 (18.52)	9 (32.14)	7 (25.93)	13 (41.93)	4.065	0.255
Diabetes	25 (22.12)	6 (22.22)	3 (10.71)	8 (29.63)	8 (25.80)	3.243	0.356
**Coronary heart disease**							
≥ 1 comorbidity	11 (9.73)	1 (3.70)	3 (10.71)	3 (11.11)	4 (12.91)	1.561	0.668
1–2	41 (36.28)	8 (29.63)	13 (46.42)	9 (33.33)	11 (35.48)	6.087	0.414
≥ 3	18 (15.93)	4 (14.81)	1 (3.57)	6 (22.22)	7 (22.58)		
**Medications**							
1–2	24 (21.24)	2 (7.40)	8 (28.57)	5 (18.52)	9 (29.03)	6.479	0.372
≥ 3	21 (18.58)	6 (22.22)	4 (14.29)	4 (14.81)	7 (22.58)		
**Symptoms of illness onset**							
Fever	65 (57.52)	12 (44.44)	13 (46.43)	18 (66.67)	22 (70.96)	6.518	0.089
Cough	56 (49.56)	11 (40.74)	12 (42.86)	13 (48.15)	20 (64.52)	4.139	0.247
Temperature (°C)	36.5 (36.4–36.8)	36.6 (36.3–36.8)	36.6 (36.4–37.0)	36.5 (36.3–37.0)	36.5 (36.4–36.7)	4.404	0.221
> 37.3	16 (14.16)	4 (14.81)	6 (21.42)c	6 (22.22)f	0 (0.00) c f	7.784	0.018*
Heart rate (beats/min)	90.0 (78.0–102.5)	96.0 (83.0–104.0)	89.5 (77.75–96.0)	84.0 (76.0–96.0)	90.0 (79.0–105.0)	3.307	0.347
> 100	29 (25.66)	8 (29.63)	5 (18.52)	6 (22.22)	10 (32.26)	1.991	0.574
SBP (mm Hg)	129.0 (118.5–140.5)	126.0 (114.0–135.0)	128.0 (114.5–143.3)	124.0 (116.0–140.0)	136.0 (126.0–150.0)	8.421	0.038*
≥ 140	33 (29.20)	3 (11.11)e	8 (28.57)	8 (29.63)	14 (45.16) e	8.101	0.044*
DBP (mm Hg)	80.0 (73.0–90.0)	81.0 (75.0–90.0)	75.5 (71.0–84.75)	78.0 (70.0–90.0)	83.0 (78.0–91.0)	7.425	0.060
≥ 90	29 (25.66)	7 (25.93)	4 (14.29)	8 (29.63)	10 (32.26)	2.830	0.419
SPO_2_ (%)	98.0 (96.0–98.0)	98.0 (97.0–99.0)a	96.5 (94.25–98.0)ac	97.0 (96.0–98.0)	98.0 (97.0–99.0)c	11.304	0.010*
Creatinine (μmol /l)	69.0 (57.0–87.0)	64.0 (54.0–83.0)	77.5 (55.0–96.3)	70.0 (57.0–87.0)	67.0 (60.0–80.0)	2.723	0.436
Urea (mmol/l)	4.4 (3.4–5.6)	4.4 (3.1–5.4)	4.6 (3.2–5.7)	4.0 (3.2–5.6)	4.7 (3.7–5.8)	2.097	0.552
**Outcome**							
Length of stay (days)	13.0 (10.0–17.0)	14.0 (10.0–18.0)	14.0 (10.0–17.0)	14.0 (9.0–18.0)	12.0 (11.0–15.0)	0.653	0.884
Developing a severe disease	2 (1.77)	1 (3.70)	0 (0.00)	0 (0.00)	1 (3.23)	1.950	0.863
Oxygen therapy	70 (61.95)	14 (51.85)	18 (64.29)	20 (74.07)	18 (58.06)	3.115	0.375

Data are shown as median (interquartile range) or n (%). *P* values were calculated by Kruskal–Wallis test, χ^2^ test or Fisher’s exact test, as appropriate. SBP, systolic blood pressure; DBP, diastolic blood pressure; SPO_2,_ pulse oximeter O_2_ saturation; * denoted *P* < 0.05.

a denoted *P*_bonferroni_ < 0.05 between group 1 and group 2.

b denoted *P*_bonferroni_ < 0.05 between group 2 and group 3.

c denoted *P*_bonferroni_ < 0.05 between group 2 and group 4.

d denoted *P*_bonferroni_ < 0.05 between group 1 and group 3.

e denoted *P*_bonferroni_ < 0.05 between group 1 and group 4.

f denoted *P*_bonferroni_ < 0.05 between group 3 and group 4.

### Comparisons of laboratory indices and radiological findings across the four groups

Comparisons of laboratory indices and radiological findings across the four groups are shown in Table 2. The level of hs-cTnI in group 2 [10.25 (IQR 6.75–15.63) ng/L] was significantly higher than those in group 1 [1.90 (IQR 1.90–8.80) ng/L] and group 4 [1.90 (IQR 1.90–5.80) ng/L] (all *P*_bonferroni_ < 0.05). The levels of myoglobin, LDH, and CRP in group 2 were significantly higher than those in group 4 (all *P*_bonferroni_ < 0.05). The levels of lymphocyte count in group 1 and group 2 were significantly lower than that in group 4 (all *P*_bonferroni_ < 0.05). Notably, the proportion of patients who had a level of hs-cTnI ≥ 5 ng/L in group 2 (85.71%) was significantly higher than those in the other three groups (37.04%, 51.85%, and 25.81%, respectively) (all *P*_bonferroni_ < 0.05). In addition, the incidence of bilateral pneumonia in group 2 (71.43%) was significantly higher than those in group 1 (33.33%) and group 4 (25.81%) (all *P*_bonferroni_ < 0.05).

**Table 2:** Comparisons of cardiac biomarkers, CRP, lymphocyte count, and radiological findings across the four groups.

Characteristics	Total (*n* = 113)	Group 1 (*n* = 27)	Group 2 (*n* = 28)	Group 3 (*n* = 27)	Group 4 (*n* = 31)	H/χ^2^	*P*
Troponin I (ng/L)	4.50 (1.90–10.60)	1.90 (1.90–8.80)a	10.25 (6.75–15.63)ac	5.30 (1.9–11.7)	1.90 (1.90–5.80)c	21.054	0.000*
≤ 34.2 (for males) ≤ 15.6 (for females) #	103 (91.15)	25 (92.59)	23 (82.14)	26 (96.30)	29 (93.55)	3.993	0.262
> 34.2 or 15.6	10 (8.85)	2 (7.41)	5 (17.86)	1 (3.70)	2 (6.45)		
Or < 5	57 (50.44)	17 (62.96)a	4 (14.29)abc	13 (48.15)b	23 (74.19)c	23.389	0.000*
≥ 5	56 (49.56)	10 (37.04)	24 (85.71)	14 (51.85)	8 (25.81)		
BNP (pg/mL)	96.00 (27.5–185.0)	47.0 (19.0–178.0)	155.5 (42.3–229.3)	178.0 (45.0–243.0)	41.0 (20.0–148.0)	9.935	0.019*
< 486 #	97 (85.84)	25 (92.59)	24 (85.71)	21 (77.78)	27 (87.10)	2.399	0.503
≥ 486	16 (14.16)	2 (7.41)	4 (14.29)	6 (22.22)	4 (12.90)		
Myoglobin (ng/mL)	36.90 (27.80–77.95)	33.2 (23.5–55.5)	68.15 (33.50–181.52)c	38.40 (26.60–78.2)	32.50 (23.30–43.70)c	13.470	0.004*
≤ 154.9 #	100 (88.50)	23 (85.19)	20 (71.43)c	26 (96.30)	31 (100.00)c	13.047	0.001*
> 154.9	13 (11.50)	4 (14.91)	8 (28.57)	1 (3.70)	0 (0.00)		
CKMB (ng/mL)	0.80 (0.50–1.20)	0.60 (0.40–1.30)	0.90 (0.60–2.08)	0.80 (0.50–1.20)	0.70 (0.50–1.10)	2.665	0.446
≤ 7.2#	112 (99.12)	26 (96.30)	28 (100.00)	27 (100.00)	31 (100.00)	0.101	1.000
> 7.2	1 (0.88)	1 (3.70)	0 (0.00)	0 (0.00)	0 (0.00)		
LDH (U/L)	204.0 (169.5–284.0)	204.0 (187.0–246.0)e	267.0 (235.0–351.3)c	224.0 (153.0–342.0)f	170.0 (155.0–195.0)cef	24.288	0.000*
135–225#	64 (56.64)	15 (55.56)e	6 (21.43)c	14 (51.85)f	29 (93.55)cef	31.595	0.000*
> 225	49 (43.36)	12 (44.44)	22 (78.57)	13 (48.15)	2 (6.45)		
Lymphocyte count (× 10^9^/L)	1.28 (0.85–1.76)	1.17 (0.86–1.66)e	1.12 (0.69–1.42)c	1.30 (0.81–1.80)	1.62 (1.31–2.14)ec	14.291	0.003*
< 1.1	40 (35.40)	12 (44.44)e	13 (46.43)c	11 (40.74)	4 (12.90)ce	9.623	0.022*
1.1–3.2 #	73 (64.60)	15 (55.56)	15 (53.57)	16 (59.26)	27 (87.10)		
CRP (mg/L)	9.80 (1.55–46.20)	12.7 (0.90–48.0)e	34.45 (6.63–64.93)c	29.0 (3.90–55.60)f	2.10 (0.50–4.40)cef	21.742	0.000*
< 10.0 #	58 (51.33)	12 (44.44)e	9 (32.14)c	9 (33.33)f	28 (90.32)cef	27.005	0.000*
≥ 10.0	55 (48.67)	15 (55.56)	19 (67.86)	18 (66.67)	3 (9.68)		
**Chest CT findings**							
Bilateral pneumonia	53 (46.90)	9 (33.33)a	20 (71.43)ac	16 (59.26)	8 (25.81)c	15.954	0.001*
Unilateral pneumonia	60 (53.10)	18 (66.67)	8 (28.57)	11 (40.74)	23 (74.19)		

Data are shown as median (interquartile range) or n (%). *P* values were calculated by Kruskal–Wallis test, χ^2^ test or Fisher’s exact test, as appropriate. BNP, B-type natriuretic peptide; CKMB, creatine kinase-myocardial band; LDH, lactic dehydrogenase; CRP, C-reactive protein; * denoted *P* < 0.05; # denoted Reference intervals of analytes.

a denoted *P*_bonferroni_ < 0.05 between group 1 and group 2.

b denoted *P*_bonferroni_ < 0.05 between group 2 and group 3.

c denoted *P*_bonferroni_ < 0.05 between group 2 and group 4.

d denoted *P*_bonferroni_ < 0.05 between group 1 and group 3.

e denoted *P*_bonferroni_ < 0.05 between group 1 and group 4.

f denoted *P*_bonferroni_ < 0.05 between group 3 and group 4.

### Comparisons of BNP, myoglobin, CRP, lymphocyte count, SpO_2_, radiological findings, and outcome between COVID-19 patients with hs-cTnI under 5 ng/L and ≥ 5 ng/L

Comparisons of BNP, myoglobin, CRP, lymphocyte count, SpO_2_, radiological findings, and outcome between COVID-19 patients with hs-cTnI under 5 ng/L and ≥ 5 ng/L are shown in Table 3. Compared with patients with hs-cTnI under 5 ng/L, those with hs-cTnI ≥ 5 ng/L presented with significantly lower lymphocyte count (*P* = 0.000) and SpO_2_ (*P* = 0.002) and higher CRP (*P* = 0.000). Patients with hs-cTnI ≥ 5 ng/L had significantly higher incidence of bilateral pneumonia (*P* = 0.000) and significantly longer hospital length of stay (*P* = 0.000). Additionally, 2 patients with hs-cTnI ≥ 5 ng/L developed a severe disease during their hospitalization, while no patients with hs-cTnI under 5 ng/L did.

**Table 3:** Comparisons of BNP, myoglobin, CRP, lymphocyte count, SpO_2_, radiological findings, and outcome between patients with hs-cTnI under 5 ng/L and ≥ 5 ng/L.

Characteristics	hs-cTnI < 5 ng/L (*n* = 57)	hs-cTnI ≥ 5 ng/L (*n* = 56)	Z/χ^2^	*P*
BNP (pg/mL)	37.0 (18.0–115.5)	175.5(70.5–300.0)	− 5.328	0.000
< 486 #	54 (94.74)	43 (76.79)	7.489	0.006
≥ 486	3 (5.26)	13 (23.21)		
Myoglobin (ng/mL)	29.00(22.85–34.75)	75.65(38.70–127.05)	− 6.593	0.000
≤ 154.9 #	57 (100.00)	43 (76.79)	14.952	0.000
> 154.9	0 (0.00)	13 (23.21)		
Lymphocyte count (× 10^9^/L)	1.59(1.20–2.05)	1.09 (0.69–1.32)	− 4.566	0.000
< 1.1	12 (21.05)	28 (50.00)	10.351	0.001
1.1–3.2 #	45 (78.95)	28 (50.00)		
CRP (mg/L)	2.20 (0.75–9.90)	33.85 (9.85–71.20)	− 5.540	0.000
< 10.0 #	43 (75.44)	15 (26.79)	26.765	0.000
≥ 10.0	14 (24.56)	41 (73.21)		
SpO_2_ (%)	98.0 (97.0–98.5)	97.0 (95.0–98.0)	3.047	0.002
**Chest CT findings**				
Bilateral pneumonia	16 (28.07)	37 (66.07)	16.380	0.000
Unilateral pneumonia	41 (71.93)	19 (33.93)		
**Outcome**				
Hospital length of stay (days)	12.0 (9.0–15.0)	15.5 (12.0–21.75)	− 4.111	0.000
Oxygen therapy	32(56.14)	38(67.86)	1.645	0.246
Developing a severe disease	0(0.00)	2(3.57)	2.072	0.243

Data are shown as median (interquartile range) or n (%). *P* values were calculated by Mann–Whitney U test, χ^2^ test or Fisher’s exact test, as appropriate. BNP, B-type natriuretic peptide; CRP, C-reactive protein; SPO_2,_ pulse oximeter O_2_ saturation; * denoted *P* < 0.05; # denoted Reference intervals of analytes.

### Correlations between cardiac biomarkers and SpO_2_, lymphocyte count, and CRP

**Figure 1:**
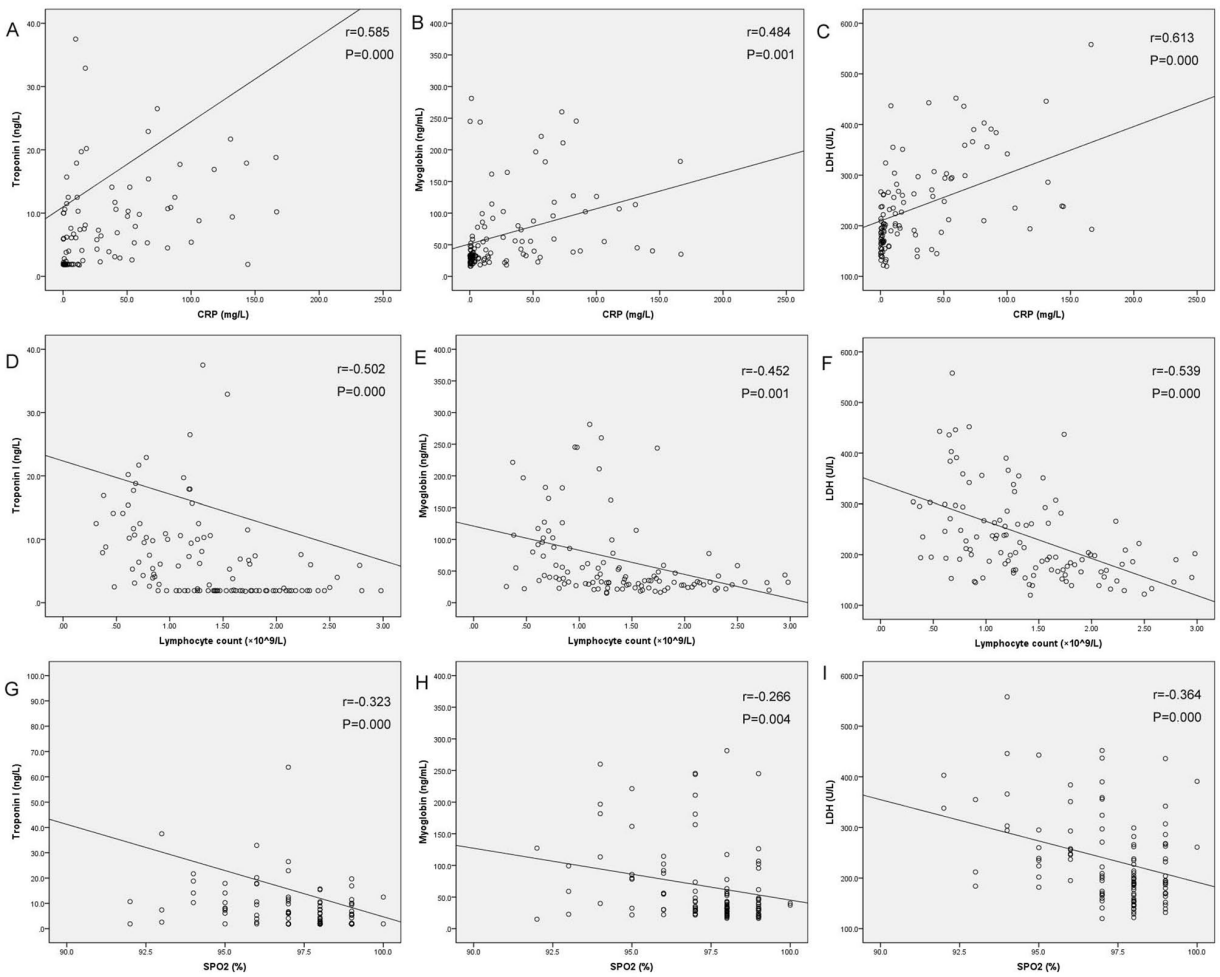
Scatter plots of cardiac biomarkers and SPO_2_, lymphocyte count, and CRP. The levels of cardiac biomarkers were significantly correlated with those of SPO_2_, lymphocyte count, and CRP. SPO_2_, pulse oximeter O_2_ saturation; CRP, C-reactive protein; hs-cTnI, high sensitivity troponin I; BNP, B-type natriuretic peptide; CKMB, creatine kinase-myocardial band; LDH, lactic dehydrogenase.

Scatter plots of cardiac biomarkers and SpO_2_, lymphocyte count, and CRP are shown in Fig. 1. The levels of hs-cTnI (Fig. 1A,D), myoglobin (Fig. 1B,E), and LDH (Fig. 1C,F) were found to be positively correlated with that of C-reactive protein but negatively correlated with that of lymphocyte count. The level of SpO_2_ was found to be negatively correlated with those of hs-cTnI (Fig. 1G), myoglobin (Fig. 1H), and LDH (Fig. 1I).

### Boxplots of cardiac biomarkers, SpO_2_, lymphocyte count, and CRP across the four groups

**Figure 2:**
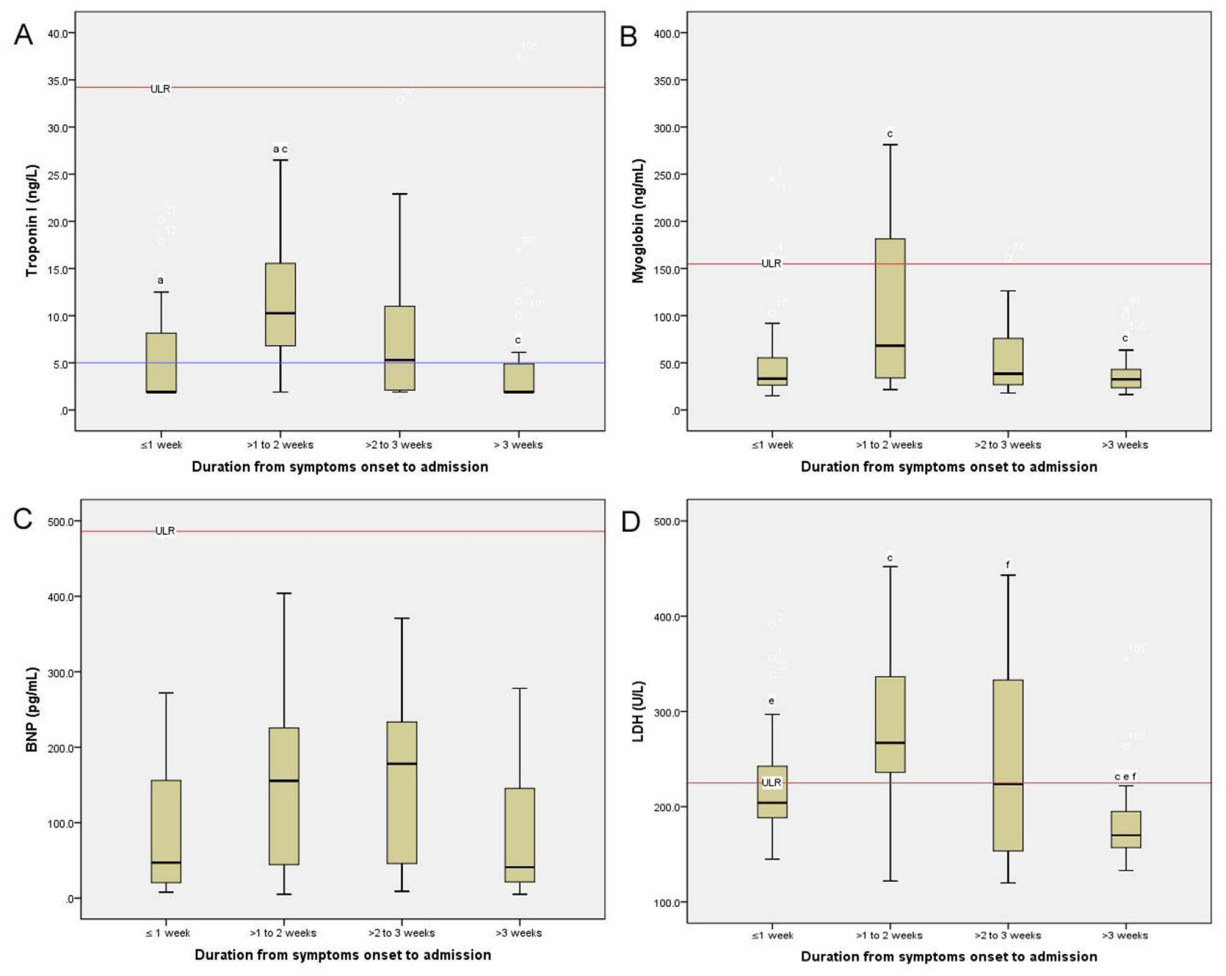
Simulative changes of cardiac biomarkers in non-severe patients with COVID-19 across the four groups. COVID-19 patients who were in the second week after symptoms onset presented the highest levels of hs-cTnI, myoglobin, BNP, and LDH. hs-cTnI, high sensitivity troponin I; BNP, B-type natriuretic peptide; LDH, lactic dehydrogenase; ULR, Upper limit of reference intervals; LLR, Lower limit of reference intervals.

**Figure 3:**
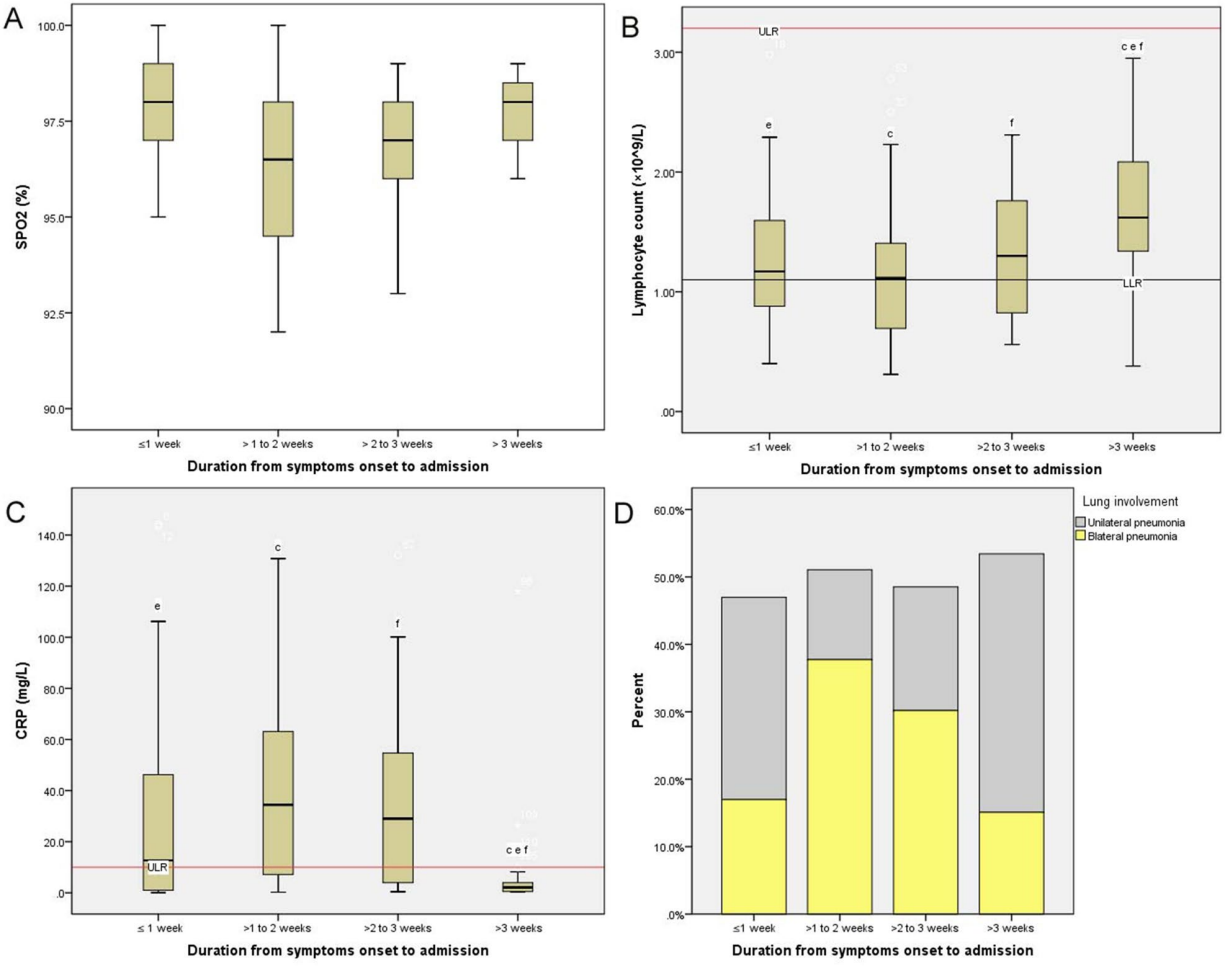
Simulative changes of SpO_2_, lymphocyte count, CRP, and radiological findings in non-severe patients with COVID-19 across the four groups. COVID-19 patients who were in the second week after symptoms onset presented the highest levels of CRP and the lowest levels of SpO_2_ and lymphocyte count. Meanwhile, the patients had the highest incidence of bilateral pneumonia. SPO_2_, pulse oximeter O_2_ saturation; CRP, C-reactive protein; ULR, Upper limit of reference intervals; LLR, Lower limit of reference intervals.

The boxplots of cardiac biomarkers, SpO_2_, lymphocyte count, and CRP across the four groups are shown in Figs. 2 and 3. Patients in group 2 had the highest levels of hs-cTnI, myoglobin, lactic dehydrogenase, CRP, and lung involvement across the four groups. In contrast, Patients in group 2 presented the lowest levels of lymphocyte and SpO_2_ across the four groups.

### Subgroup analysis

When the patients with higher troponin levels than 99th percentile were excluded, the level of hs-cTnI in group 2 [1.9 (IQR 1.9–5.0) ng/L] was still significantly higher than those in group 1 [1.90 (IQR 1.825–2.650) ng/L] and group 4 [1.90 (IQR 1.90–5.35) ng/L] (all *P*_bonferroni_ < 0.05). Similarly, the proportion of patients who had a level of hs-cTnI ≥ 5 ng/L in group 2 was also significantly higher than those in the other three groups (all *P*_bonferroni_ < 0.05). Consistent with the aforementioned results, compared with patients with hs-cTnI under 5 ng/L, those with hs-cTnI ≥ 5 ng/L presented significantly lower lymphocyte count and SpO2, higher CRP and incidence of bilateral pneumonia, and longer hospital length of stay (all *P*_bonferroni_ < 0.05).

## Discussion

In this study, we observed that non-severe patients with COVID-19 were most likely to suffer cardiac damage in the second week after symptoms onset. During this period, a detectable level of hs-cTnI ≥ 5 ng/L might be a remarkable manifestation of early cardiac damage in the patients. To the best of our knowledge, this is the first study to demonstrate the value of a lower cutoff threshold of hs-cTnI than the 99th percentile in identification of early cardiac damage in non-severe patients with COVID-19.

In this study, COVID-19 patients in the second week after symptoms onset had the highest levels of cardiac biomarkers. This result is in accord with Zhou et al.’^8^ study indicating that the level of hs-cTnI increased significantly from 10 to 13 days after symptoms onset in COVID-19 patients. Similar to the results of a longitudinal study^17^, the patients in the second week after symptoms onset had the highest levels of CRP and LDH, which could be considered as the signs of the systemic inflammatory response. Moreover, in line with a previous study^3^, our results demonstrated that the cardiac biomarkers were highly related to lymphocyte count and CRP, suggesting that cardiac damage of COVID-19 was related to viral response and hyperinflammation. Accordingly, non-severe patients with COVID-19 in the second week after symptoms onset were most likely to suffer cardiac damage.

Interestingly, patients in group 2 had a significantly higher level of hs-cTnI compared with patients in either group 1 or group 4. In particular, a remarkably higher proportion of patients who had a level of hs-cTnI ≥ 5 ng/L was also observed in this group compared with the other three groups. There are several explanations for this result. First, cardiac troponin is theoretically released from cardiomyocytes following reversible injury, irreversible injury, or apoptosis, and a significant increase in circulation concentration following the release of the protein by injured cardiomyocytes^18,19^. Given this theory and the data which suggested that patients with suspected acute coronary syndrome who had a detectable level of hs-cTnI ≥ 5 ng/L are at high risk of cardiac ischemic events (e.g. sevenfold greater risk of subsequent myocardial infarction in Bularga et al.’s study)^12,13^, a detectable level of hs-cTnI ≥ 5 ng/L could also be considered as a sign of the high risk of cardiac damage. Second, the aforementioned proportion in group 2 (89%) was higher than those in the general population (less than 25%)^20^, in patients with chronic obstructive pulmonary disease (less than 40.52%)^21^, or even in patients with suspected acute coronary syndrome (55.65%)^12^. In this way, a detectable level of hs-cTnI ≥ 5 ng/L could be considered as a manifestation of cardiac damage. Third, our results showed that non-severe patients with COVID-19 who had a level of hs-cTnI ≥ 5 ng/L had significantly higher levels of CRP and lung involvement and lower levels of SpO_2_ and lymphocyte count compared with those with hs-cTnI under 5 ng/L. Meanwhile, it was suggested that high levels of CRP and lung involvement, as well as low levels of oxygen saturation and lymphocyte count, were highly related to cardiac injury in COVID-19 patients^20–22^. Hence, a relatively high level of hs-cTnI release (≥ 5 ng/L but < 99th percentile) might be a sign of the high risk of cardiac damage in non-severe patients with COVID-19.

Of note, the hospital length of stay was found to be significantly longer in the patients with hs-cTnI ≥ 5 ng/L in the present study. Similarly, half of the 99th percentile for hs-cTnI was reported to be significantly related to the fatal outcomes in patients with COVID-19^10,23^. Thus, the cutoff of hs-cTnI in stratifying cardiac damage in non-severe patients with COVID-19 was recommended to be redefined, and the cutoff threshold of abnormality for hs-cTnI was suggested to be lower than the 99th percentile.

Our findings could help us to fully understand the cardiac damage caused by SARS-Cov-2, providing important implications for cardiovascular management in patients with COVID-19. To identify early cardiac damage in patients with COVID-19, monitoring cardiac biomarkers (particularly hs-cTnI) aggressively was recommended to be considered seriously during treatment, especially when the patients are in the second week after symptoms onset. More importantly, using the standard cutoff of hs-cTnI (99th percentile) might underestimate the extent of cardiac damage in non-severe patients with COVID-19, and the cutoff of hs-cTnI should be redefined (e.g. ≥ 5 ng/L) to identify patients who are at high risk of cardiac damage.

### Limitation

Despite the intriguing findings of this study, several important limitations should be taken into account. First, although we have considered several covariates, other potential confounders have not been fully adjusted. Second, recall bias regarding the date of symptoms onset was great due to the long duration, and misclassification of disease course may exist in some patients. Third, The underlying structural heart diseases were not completely ruled out to be coexisting with COVID-19 patients in this study, and thereby whether elevated levels of cardiac biomarkers in COVID-19 patients were caused by the potential coexisting heart diseases is still unknown. Additionally, the level of troponin was found to be related to BMI and epicardial adipose tissue in COVID-19 patients^24^. However, the data regarding BMI and epicardial adipose tissue were not recorded in this study. The potential imbalance in these data across the four groups would have an impact on the results, and the conclusion was suggested to be interpreted with caution. Fourth, it is thought that the neutrophil to lymphocyte count ratio is superior to the lymphocyte count in evaluating the disease. However, as the data of neutrophils were missing, the neutrophil to lymphocyte count ratio was not applied in this study. Last but not least, our study is single-centered research with a small sample size, and it may be underpowered to detect a significant difference between patients with different durations at presentation. A large prospective cohort study is needed to verify our conclusions in the future.

## Conclusions

In conclusion, non-severe patients with COVID-19 in the second week after symptoms onset were most likely to suffer cardiac damage. A lower cutoff threshold of hs-cTnI than the 99th percentile (e.g. 5 ng/L) was suggested to identify early cardiac damage at presentation in the patients. Thus, using the standard cutoff of hs-cTnI (99th percentile) might underestimate the extent of cardiac damage in patients with COVID-19, and the cutoff threshold (e.g. ≥ 5 ng/L) of abnormality for hs-cTnI was suggested to be lower than the 99th percentile. Our results can help to provide important references for the management and enrollment for future prospective studies to accurately validate the risk stratification of cardiac involvement in non-severe patients with COVID-19.

## Data Availability

The datasets used and/or analyzed during the current study are available from the corresponding author on reasonable request.
